# A contemporary systematic review on deterministic numerical simulations of light propagation in head tissues

**DOI:** 10.1007/s12551-025-01403-w

**Published:** 2026-01-19

**Authors:** Filipa Fernandes, Nuno Sousa, Filipe S. Silva, Óscar Carvalho, Susana O. Catarino

**Affiliations:** 1 Center for Micro-ElectroMechanical Systems (CMEMS-UMINHO), University of Minho, Guimarães, Portugal; 2 Centro Universitário Max-Planck (UniMAX), Indaiatuba, SP Brazil; 3 Centro Universitário de Jaguaríuna (UniFAJ), Jaguaríuna, SP Brazil; 4 LABBELS—Associate Laboratory, Braga/Guimarães, Portugal

**Keywords:** Brain, Light propagation, Near-infrared spectroscopy, Numerical simulations, Photobiomodulation, Photon propagation

## Abstract

Understanding how light interacts with the head’s tissues is relevant for several biomedical applications. Since in vivo studies involve ethical considerations, numerical simulations have become a recognised alternative method for studying light propagation in biological tissue. Although Monte Carlo methods are the gold standard for these studies, deterministic simulations are becoming more common due to their lower computational cost. Thus, this document reviews articles published after 2010, containing deterministic numerical simulations of light propagation (visible to infrared wavelengths) in human head tissues, to define how these methods are implemented and whether they are a viable alternative to the stochastic Monte Carlo algorithms. Most of the selected articles included a 3D simulation, using Finite Element Methods (FEM) to solve the Diffusion Equation (DE), with Robin boundary conditions, and considering the tissues as horizontal rectangular layers, to improve imaging techniques’ algorithms. Regarding target areas, there is an almost identical number of records studying the brain as a whole or dividing it into grey and white matter, while more studies consider the scalp and skull as individual layers instead of grouping them. The cerebrospinal fluid (CSF) was included in more than half of the studies, confirming that it is possible to simulate this tissue using the DE, if the optical parameters are adequate. Some of the challenges identified in the reported simulations are the variations in the optical properties of tissues (reduced scattering and absorption coefficients) and oversimplifications of the geometric models, which raise the question of whether using subject-specific data could improve the outcomes of light-based diagnosis and therapies. Although Monte Carlo methods are still the most commonly used for the simulation of optical properties, all the reviewed works reached comprehensive results, with most of them showing that deterministic numerical simulations can be an efficient and relatively accurate alternative to the time-consuming Monte Carlo methods.

**Supplementary Information:**

The online version contains supplementary material available at 10.1007/s12551-025-01403-w.

## Introduction

The study of how light interacts with biological tissue is a topic of great interest in the medical field, since it allows the development and improvement of technologies for the diagnosis, monitoring, and treatment of different pathologies.

In particular, near-infrared (NIR) light is used across a range of technologies applied at different stages of medical intervention, which can provide functional and anatomical data that is useful for different neurological applications. For instance, optical coherence tomography (OCT) is a non-invasive technique which, in most devices, uses NIR light due to its deeper penetration in biological tissues, providing cross-sectional imaging of the tissue structure (Arridge 1999; Shu et al. 2017; Aumann et al. 2019). Although it is widely used for ophthalmology purposes, recent research has been trying to correlate retinal data obtained from OCT to the diagnosis of dementia-related conditions, such as Alzheimer’s and mild cognitive impairment (Thomson et al. 2015; Moran et al. 2022).

Near-Infrared Spectroscopy (NIRS) is another non-invasive technology, based on the differences in absorption of NIR light between oxygenated and deoxygenated haemoglobin (Hamaoka and McCully 2019). It is commonly used for bedside monitoring of cerebral oxygenation, particularly during the perioperative period of cardiac interventions (Scheeren et al. 2012; Ali et al. 2022). When NIRS technology is used for functional mapping of brain activity, it can be referred to as functional NIRS (fNIRS). fNIRS is particularly used for task-related studies, as it allows the detection of the activation of specific brain regions according to variations in cerebral oxygenation (Chen et al. 2020; Peng and Hou 2021). It is also commonly applied in paediatric care and research, as it is convenient and simple to use in neonates and infants, and their relatively thinner head tissues allow for better NIR light penetration (Gervain et al. 2023). A more advanced application of fNIRS is diffuse optical tomography (DOT), which uses the same NIRS principles, but employs a three-dimensional (3D) image reconstruction of cerebral oxygenation patterns (Vesoulis et al. 2021; Vidal-Rosas et al. 2023).

Finally, regarding the application of NIR light as a therapy, currently referred to in the literature as photobiomodulation (PBM), it has been studied for a wide range of applications, such as wound healing (Kuffler 2015), to improve dental implant success (Dompe et al. 2020), for oral mucositis (Hanna et al. 2020), and, more related to the topic of this research, for neurological disorders (Fernandes et al. 2024). Specifically, for neurological pathologies, when light (in wavelengths between 600 and 1100 nm) is applied to the scalp or intranasally to reach the brain tissue, it is referred to as transcranial PBM (tPBM). It is a non-invasive therapy that has neuromodulatory effects in the tissues, which overall promote cell regeneration and increase oxygenation (Hennessy and Hamblin 2018; Salehpour et al. 2018). This therapy resulted in the improvement of patients’ symptoms in several clinical studies for different neurological conditions. For dementia-related pathologies, studies showed that patients improved cognition and behaviour, without negative side effects (Chao 2019; Salehpour et al. 2019; Spera et al. 2021). In traumatic brain injury clinical studies, patients showed improved cognition and reaction times, which relate to better recovery outcomes (Grover et al. 2017; Hipskind et al. 2018; Figueiro Longo et al. 2020). Additionally, in Parkinson’s disease studies, patients self-reported improvements and showed better results in neuropsychological assessment tests, and improvements in mobility and fine motor skills (Hamilton et al. 2019; Liebert et al. 2021).

Although all of these applications appear to be effective and useful in a medical setting, there are still limitations noted in the literature. For NIRS-based technologies, these include noise signals from motion artifacts and physiologic sources, and the complexity of signal processing and interpretation (Chen et al. 2020; Ali et al. 2022). For tPBM therapies, limitations are based on the uncertainty of optimal light parameters for stimulation, such as wavelength and power density (Fernandes et al. 2024).

Therefore, characterising the interaction of NIR light with the head tissues is fundamental for improving algorithms for image processing in the abovementioned imaging and monitoring applications, as well as determining efficient and safe dosimetry for therapeutic applications, such as tPBM. Computational simulations of light propagation in the head tissues are an effective solution to study several scenarios and predict outcomes, without compromising the patients’ safety and time, or the ethical considerations of animal studies.

Monte Carlo methods are the gold standard for light simulation studies in biological tissues, with a stochastic approach for modelling photon interaction with biological tissue, based on the probability of movement and interaction of photons with the environment (Periyasamy and Pramanik 2017; Tang and Yao 2021), although being able to produce accurate results, Monte Carlo methods show low computational efficiency, resulting in higher simulation times (Sultan et al. 2018; Hirvi et al. 2023). Deterministic numerical methods, such as the Finite Element Method (FEM), are emerging as an alternative, showing equivalent results while requiring fewer processing resources, which translates into reduced computational times. Particularly, when studying light interaction with biological tissue, deterministic methods are used to solve partial differential equations (PDEs) to determine photon interaction with the tissues.

Since computer simulation is a constantly evolving field, this article aims to perform a contemporary literature review of studies reporting deterministic numerical methods for light propagation in the head tissues, covering the last 15 years, to provide an updated view on how these simulations are implemented. From the type of deterministic methods used to solve the equations governing light propagation, to the morphology and optical properties of the head tissues, current trends and existing challenges will be considered, and the most common fields of application for these simulations will be explored. Finally, it will be proposed whether these techniques can produce reliable results while overcoming the drawbacks of the Monte Carlo approaches.

### Theoretical background

Different mathematical models can describe light propagation through a material. For example, Maxwell’s equations represent light as an electromagnetic wave, offering an accurate physical description (Sheu et al. 2012). However, in scattering media, the Radiative Transfer Equation (RTE) is generally more commonly used. The RTE works as a conservation law that considers photon absorption, scattering, and emission (Arridge and Schotland 2009; Bazrafkan and Kazemi 2014; Hirvi et al. 2023).

Despite its accuracy, the RTE is complex to solve, and for this reason, most studies consider the Diffusion Approximation (DA) as an alternative to describe photon transport in biological tissues, provided that certain assumptions are met (Yaroslavsky et al. 1995; Venugopalan et al. 1998; Tschudi 2008). Considering the absorption coefficient (*µ*_*a*_) and the reduced scattering coefficient (*µ*_*s*_*’*) of a certain medium, these assumptions include that the relation *µ*_*a*_/*µ*_s_’ < < 1 is fulfilled, that after sufficient scattering the radiance is nearly isotropic in the medium, and that the photon mean free path must be much smaller than the total propagation distance under study (Wang and Wu 2007; Re et al. 2025). It should be noted that even if the previous condition is met, the DA shows some issues in void-like structures, which, for the present research, means the cerebrospinal fluid (CSF). Nevertheless, and as will be noted in further sections, recent research was able to provide accurate results for this tissue using the DA (Kannan and Przekwas 2012; Vera et al. 2020; Lohrengel et al. 2021).

Equation 1 relates to a representation of the diffusion equation (DE), a PDE which results from the DA of the RTE, where *c* is the light speed in the medium, $\phi \left(\overrightarrow{r},t\right)$ the photon fluence rate (total light energy density), *D* is the diffusion coefficient $\left(D=\frac1{3\left(\mu_a+\mu_s'\right)}\right)$, which depends on the absorption and reduced scattering coefficients, and $S(\overrightarrow{r},t)$ represents the source term (Martelli et al. 1999; Wang and Wu 2007; Re et al. 2025).

1 $$\begin{array}{c}\frac{1}{c}\frac{\partial \phi \left(\overrightarrow{r},t\right)}{\partial t}-D{\nabla }^{2}\phi \left(\overrightarrow{r},t\right)+{\mu }_{a}\phi \left(\overrightarrow{r},t\right)=S\left(\overrightarrow{r},t\right)\end{array}$$

Considering a steady-state regime, where $\phi$ does not change over time, the DE can be transformed into a form resembling the Helmholtz equation, which is shown in Eq. 2 (Takatani and Graham 1979; Helton et al. 2022; Dhmiri et al. 2023).

2 $$\begin{array}{c}-D{\nabla }^{2}\phi \left(\overrightarrow{r}\right)+{\mu }_{a}\phi \left(\overrightarrow{r}\right)=S\left(\overrightarrow{r}\right)\end{array}$$

The solution of the DE in biological tissues may be accomplished using different methods. The focus of this research is the use of deterministic numerical methods to solve these equations, or equivalent, and understanding how these algorithms compare with the most widely used Monte Carlo methods, which provide a stochastic approach to the solution of the full RTE.

#### Stochastic numerical simulations

Monte Carlo simulations follow a stochastic approach to modelling photon interaction with biological tissues, by finding a numerical solution for the RTE using random sampling (Fang 2010). Typically, in such simulations, the individual trajectory of a photon is considered as a random event, governed by its probability of being scattered, absorbed, refracted, or reflected, which in turn depends on the tissues’ optical properties (Krasnikov et al. 2019). The simulation continues tracking each photon until it is either absorbed or exits the tissue (Zołek et al. 2006). A statistical analysis of a large number of photon trajectories provides an estimate of photon transport that converges towards a realistic representation of these phenomena. The most notable benefit of Monte Carlo simulations is their ability to produce accurate results in complex media; however, this accuracy comes with a high computational cost, since it is directly related to the number of photons considered, which can range from thousands to several millions (Wang and Wu 2007; Young-Schultz et al. 2019; Jacques 2023). Nonetheless, some Monte Carlo studies apply discretization methods to improve computational efficiency; for example, the use of meshes for discretization of complex geometries (Fang 2010; Shen and Wang 2010).

#### Deterministic numerical simulations

Deterministic simulations model light propagation through biological tissues using fixed optical properties and parameters, which are the input for mathematical equations, instead of considering the photon movement as a random event. In this way, these simulations produce predictable and repeatable results, which depend only on the initial properties and governing equations. For these simulations, the DE is usually solved using discretization methods, such as the FEM. This approach facilitates the solution of the DE in complex and heterogeneous geometries, making deterministic simulations generally more computationally efficient when compared to Monte Carlo methods, which are also prone to statistical noise (Williams et al. 2003; Caron et al. 2015). Although these methods bring forth certain advantages, they are only valid if the aforementioned DA assumptions are met, which limits their applicability.

## Methods

Following the Preferred Reporting Items for Systematic Reviews and Meta-Analyses (PRISMA) guidelines, an extensive set of articles was collected. From that list, the articles that were relevant to the study’s focus and fitted the eligibility criteria were selected (Liberati et al. 2009).

### Data sources, search strategy, and eligibility criteria

A comprehensive electronic search was conducted using the PubMed, Scopus, and Web of Science databases for articles that described a numerical method to simulate light propagation in human head tissues. The search was carried out until October 18th, 2024, in each database, and the keywords used are detailed in Supplementary Table 1. Additionally, on that day, the Google Scholar database was used to check for any articles that might have been left out. The Microsoft® Excel software was used to export the records obtained from the search, and a software feature was used to find duplicate records, which were then eliminated following manual verification. After reviewing the remaining records’ titles, abstracts, and keywords, those that did not use simulations or did not relate to the study of the optical properties of head tissues were excluded. The remaining records were thoroughly analysed, since from the title, abstract, and keywords, they either appeared to fulfil the eligibility criteria or it was not clear if they could be included or not. Thus, after this analysis, the articles were identified according to the following eligibility criteria.

- Inclusion criteria: using a deterministic numerical method for the simulation; studying head tissues (i.e. scalp, skull, CSF, and brain); simulating light (e.g. photon interaction with tissues, electromagnetic wave propagation) between the visible and infrared spectrum.
- Exclusion criteria: reviews, conference papers, books, letters to editors; studies not available in English; simulations that did not study the human brain; cellular-scale studies; studies that did not include a light/optical simulation; simulations that did not use a numerical method; or optical imaging studies that did not describe a numerical model for the simulation (e.g. focusing on image reconstruction, noise reduction, and improvements in the beams of light).

### Data collection

To collect information from the selected articles, it is important to first understand and define which parameters are the most relevant to characterise each numerical simulation. For the current study, these parameters are as follows:

- Deterministic numerical methods used to solve the abovementioned equations, including discretization parameters (resulting in approximate solutions for the problem);
- Photon/light propagation equations to describe how the photons propagate through the defined environment (space and time-dependent);
- Boundary conditions to define how the photons behave at the boundaries of the simulation domain or the interfaces between different materials;
- Source and detector conditions to characterise the initial photon beam and its distribution until the detector point;
- Material models that represent the environment being studied, in this instance, the head tissues. For the simulation, both the optical properties of the tissues and their sizing/geometry should be defined.

From each of the included articles, the data retrieved was as follows: the software used to perform the simulation; details about the modelling conditions, namely, numerical method, light propagation model, and boundary conditions; the light parameters, such as source description, wavelength, and source/detector placement; tissue information, specifically, geometry, thickness, and optical properties (*µ*_*s*_*’*, *µ*_*a*_, anisotropy factor (*g*), and refractive index (*n*)); followed by the mesh used; any model simplifications described in the record; the application of the study in the medical field; and finally, when provided, the method of validation of the deterministic method, as well as the comparative results. When information was missing, the parameter was marked as not reported (NR).

## Results

### Search and selection of studies

**Fig. 1:**
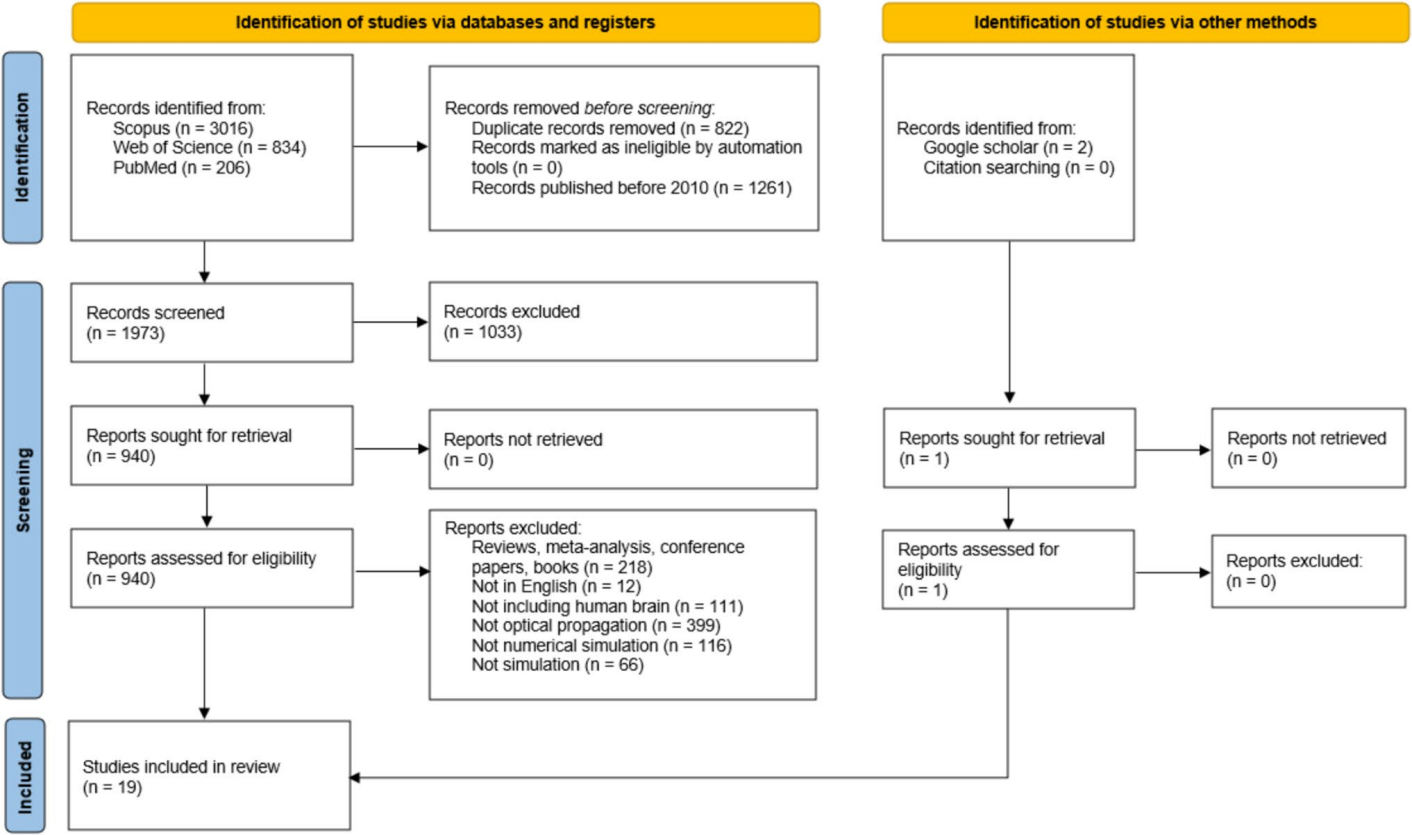
Preferred Reporting Items for Systematic Review and Meta-Analysis (PRISMA) flowchart of included and excluded studies

A total of 4056 records were retrieved from the Scopus, Web of Science, and PubMed databases. Afterwards, 822 duplicate records and 1261 articles published before 2010 were excluded, and 1973 articles were screened. From these, 1033 records were excluded for not being relevant to the research at hand. A total of 940 records were thoroughly analysed. Eighteen articles were ultimately selected according to the eligibility criteria. From the Google Scholar database, one additional article was selected. Thus, a total of 19 articles were included in this review, with Fig. 1 depicting the search and selection process resulting from this search.

### Structuring of acquired data

For the systematisation of the collected data, Table 1 was created, in which each entry relates to one of the selected articles, in chronological order, and the collected data is presented in the subsequent columns. If the article had multiple types of simulation, only the simulation that fitted the inclusion criteria was included.

**Table 1:** Overview of records’ data

Ref	Year	Software	Numerical method	Light propaga-tion model	Boundary conditions	Light parameters			Tissues			Mesh	Simplifications	Application	Validation	
						**Description**	**Wavelength (nm)**	**Source/detector placement**	**Geometry**	**Thickness (mm)**	Optical properties (µs'; µa; g; n)*				**Method**	**Conclusions**
(Wang et al. 2010)	2010	COMSOL Multi-physics	FEM	DE	Robin BC- at the surface. Neumann BC- at the interface between adjacent layers	1 mm diameter	805	8 sources and 8 detectors. Placed on the surface of the model. SDD of 30 mm	3D—5 rectangular layers	**Scalp**- 5. **Skull**- 5; 10; 20 (uniform thickness); 5–10; 5–20 (linearly varying thickness). **CSF**- 0; 1; 3; 6 (uniform thickness); 1—3; 1–6 (linearly varying thickness). **GM**- 3.5. **WM**- 15.5–36.5	**Scalp**- 0.73; 0.03; NR. **Skull-** 1.8; 0.012; NR. **CSF**- 0.3; 0.002; NR. **GM-** 2.3; 0.036; NR. **WM**- 9.1; 0.014; NR; **Active region**- 2.3; 0.052; NR	100,000 elements	NR	Imaging	NR	NR
(Kannan and Przekwas 2012)	2012	CoBi	FVM	DE	Fresnel equations	NR	690	6 detectors. Source in the front of the forehead and left side of the head	2D—slice of head (MRI). 3D—head model	NR	**Scalp and meninges**- 0.66; 0.0191; NR. **Skull-** 0.86; 0.0136; NR. **CSF**- 0.01; 0.0026; NR. **GM**- 1.11; 0.0186; NR. **WM**- 1.11; 0.0186; NR	**2D—**122 000 elements. **3D—**873 000 elements	Imitates experimental setup by using forward simulation results as pseudo-experimental data	Imaging (NIRS)	NR	NR
(Tanifuji et al. 2012)	2012	NR	FDTD	DE	Robin BC	Isotropic source	NR	Placed at a depth of 1/µs'. SDD up to 60 mm	2D—4 rectangular layers	**Scalp**- 14. **Skull-** 4. **CSF**- 2. **Brain**- 10	**Scalp**- 6; 0.005; NR; 1.56. **Skull**- 2.5; 0.025; NR; 1.56. **CSF**- 0; 0.02 or 0.001; NR; 1.46. **Brain**- 2; 0.04; NR; 1.56	NR	NR	Imaging (NIRS)	Compared with Hybrid Radiosity-Diffusion and MC	Results agree reasonably well between methods
(Sultan et al. 2013b)	2013	COMSOL Multi-physics	FEM	Helmholtz equation	Robin & Dirichlet BC	NR	680 nm, 30 to 1000 MHz	Perpendicular source and detector placement. SDD of 5 and 8 mm	2D—1 rectangular layer	NR	NR	Element size of 80 nm around source and detector, and 1000 nm elsewhere	Only one tissue	Imaging (fNIRS)	Compared with analytical solution and experimental data (solid phantoms analysis)	Small errors between FEM and experimental data. Low percentage of errors shows that FEM can replace analytical solution
(Guan et al. 2013)	2013	MATLAB	UDDO	RTE	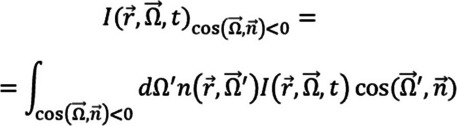	NR	NR	NR	2D—Shepp-Logan phantom	NR	**Brain**—5.8*; 0.035; 0.8; 1.3 to 1.56 for uniform n, and equation for gradient n	I × J = 256 × 256 grid regions, step of 0.02 in x and y axis	NR	Imaging (OT)	Compared with experimental data (brain phantom image with a void-like region and tumour)	Experimental measurements agree with theoretical predictions
(Sultan et al. 2013a)	2013	COMSOL Multi-physics	FEM	Helmholtz equation	Robin & Dirichlet BC	Transmission- scalp and skull; semi-transmission and reflection- cortex	680, 795, 850	Source placement: transmission and reflection- centre; semi-transmission- 10 mm from edge. perpendicular source and detector placement. SDD of 5 and 8 mm	2D; 3D—1, 2 or 3 rectangular layers	NR	**Scalp**- 1.3; 0.015; NR. **Skull-** 0.8;0.012; NR. **Brain**- 1.5; 0.035; NR	Element size of 80 nm around source and detector, and 1000 nm elsewhere	Does not consider CSF. Tissues represented with two perpendicular planes. Planar surfaces	Imaging (fNIRS)	Compared with analytical solution and experimental data (solid phantoms analysis)	Analytical and 2D FEM error: less than 4%. 2D and 3D FEM error: less than 2%. 3D FEM and experimental data error: less than 5%
(Bazrafkan and Kazemi 2014)	2014	COMSOL Multi-physics	FEM	DE	Robin BC	Source considered as delta function in time	NR	Placed at a depth of 1/µs' from the boundary	2D—sagittal head slice (MRI)	NR	**Scalp and Skull**- 0.86; 0.019; NR. **CSF**- 0.7; 0.004; NR. **Brain**- 1.11; 0.01; NR	Triangular mesh—6338 nodes, 12 576 elements	NR	Imaging (NIRS)	NR	NR
(Yue and Humayun 2015)	2015	COMSOL Multi-physics	FEM	DE	Robin BC	Gaussian beam. 4 mm diameter	850	227 sources. Source distance of 20 mm	3D—head model (Colin27 atlas)	NR	**Scalp**- 1.8; 0.012; NR. **Skull**- 1.6; 0.025; NR. **CSF**- 0.8; 0.009; NR. **GM**- 0.9; 0.036; NR. **WM**- 1.1; 0.014; NR	NR	Modified properties of CSF for DA. Did not consider blood flow	tPBM	Compared with MC	Difficult quantitative comparison. Qualitative comparison shows methods are similar
(Placati et al. 2016)	2016	MATLAB + BrainSuite (voxel)	FVM	Hybrid Radiosity-Diffusion Equation	Robin BC	Isotropic source	NR	NR	3D—head model (MRI)	NR	**Scalp**- 1.9; 0.018; NR. **Skull-** 1.6; 0.016; NR. **CSF**- 0; 0.001; NR. **GM**- 2.2; 0.036; NR. **WM**- 9.1; 0.014; NR	Cubic voxels—resolution of 0.94 mm × 0.94 mm × 1.20 mm	NR	Imaging (DOT)	Compared with MC	Improvement in computational time and accuracy
(Nourhashemi et al. 2016)	2016	COMSOL Multi-physics	FEM	Pennes’ Bio-heat Equation + Diffusion equation	Robin & Dirichlet BC	Gaussian beam. 1 mm diameter	800	Placed orthogonally at the surface centre	3D—6 cylindrical layers of adult or neonatal head	**Scalp**- 5 or 0.5. **Fat**- 1.4. **Skull-** 7 or 2. **Dura**- 0.5. CSF- 2 or 0.5. **Brain**- 80	**Scalp**- 2.358; 0.052; NR. **Fat-** 1.341; 0.011; NR. **Skull**- 1.635; 0.011; NR. **Dura**- 1.261; 0.07; NR. **CSF**- 0.32; 0.001; NR. **Brain**- 1.134 or 0.842; 0.09 or 0.008; NR	Tetrahedral mesh—1 564 530 or 1 024 918 elements. Refined mesh around source of 0.01 mm	Does not account directionality of blood perfusion. Simple geometry	Thermal distribution	Compared with MC	Relatively small systematic differences between methods
(Sultan et al. 2018)	2018	COMSOL Multi-physics	FEM	Helmholtz equation	Robin & Dirichlet BC	NR	670 nm, 100 and 1000 MHz	SDD of 20 mm	3D—3 cylindrical layers (5 to 20 mm wide). Occlusion in cortex	**Scalp**- 3. **Skull**- 4. **Brain**- 35. **Occlusion**- 2	**Scalp**- 1.3; 0.015; NR. **Skull-** 0.8; 0.012; NR. **Brain**- 1.5; 0.035; NR. **Occlusion**- 1.8; 0.06; NR	Element size of 80 nm around source and detector, and 1000 nm elsewhere	Does not consider CSF	Imaging (fNIRS)	Compared with experimental data (solid phantoms analysis)	Error between experimental and simulation results is less than 4%
(Bhattacharya and Dutta 2019)	2019	COMSOL Multi-physics	FEM	DE	Robin BC	500 mW/cm^2^	630, 700, 810	Source at the top of the head	3D—4 layer head model (Colin27 atlas)	NR	**630 nm: Scalp and Skull**- 0.858; 0.019; 0.89; NR. **CSF**- 0.25; 0.004; 0.89; NR. **GM**- 0.99; 0.127; 0.89; NR. **WM**- 4.356; 0.0661; 0.89; NR **700 nm**: **Scalp and Skull**- 0.9; 0.013; 0.89; NR. **CSF**- 0.25; 0.004; 0.89; NR. **GM**- 0.88; 0.0629; 0.89; NR. **WM**- 4.356; 0.0325; 0.89; NR **810 nm: Scalp and Skull**- 0.76; 0.016; 0.89; NR. **CSF**- 0.25; 0.0026; 0.89; NR. **GM**- 0.746; 0.0571; 0.89; NR. **WM**- 4.07; 0.0208; 0.89; NR	Delaunay tetrahedralization—58 131 nodes, 166 792 triangular elements, 917 075 tetrahedral elements	Diffuse reflection effects at the skin were excluded	tPBM and Thermal distribution	Compared with MC	Possible erroneous results for the CSF when using the DE
(Zhou et al. 2019)	2019	COMSOL Multi-physics	FEM	Pennes’ Bio-heat Equation	1- Axial symmetry: thermal insulation; 2- CSF surface: natural convection; 3- Interior boundaries: continuity; 4- Other boundaries: Dirichlet BC	Gaussian beam	808	Source at the left corner on the surface of CSF	2D- 3 cylindrical layers	**CSF**- 0.5. **GM**- 2.5. **WM**- 2.5	**CSF**- 0.066; 0.001; NR; 1.33. **GM**- 7.7; 0.025; NR. **WM**- 36.45; 0.005; NR	NR	NR	Thermal distribution	NR	NR
(Vera et al. 2020)	2020	MATLAB	FEM	DE	Robin BC. One model had planar boundaries between layers and another had one non-planar boundary (described by parametric sinusoid). No internal reflections between layers	Isotropic source	785	Placed at a depth of 1/µs'. SDD of 25 mm	2D—3 rectangular layers	**Scalp and Skull**- 9. **GM**- 11. **WM**- 50	**Scalp and Skull**- 1.09; 0.013; NR; constant. **GM-** 0.91; 0.0054; NR; constant. **WM**- 1.78; 0.024; NR; constant	Triangular mesh—19 831 nodes, 39 397 elements	Does not consider CSF. Assumed constant refraction index in the entire domain—no internal reflection between layers	Imaging (NIRS)	Compared with experimental data (solid phantoms analysis)	Experimental data showed non-planar boundaries only work with well-defined geometry. Simulations showed they are not worthwhile
(Lohrengel et al. 2021)	2022	NR	FEM (Lagrangian)	DE	Robin BC	Isotropic source	800	Placed at a depth of 1/µs'	2D—4 concentric layers; 3D—neonatal head model with 5 layers (MRI and CT)	**Scalp**- 5. **Skull**- 5. **CSF**- 2. **Brain**- 48. **Inclusion**- 10	**Scalp**- 1.9; 0.018; 0.9; NR. **Skull**- 1.6; 0.016; 0.9; NR. **CSF**- 0.032 or 0.219 or 0.406; 0.0041 or 0.0055 or 0.0069; 0.9; NR. **Brain**- 0.75; 0.043; 0.9; NR. **Inclusion-** 3; 0.1; 0.9; NR	**2D**—Triangular mesh ~ 11 200 nodes, 22 000 elements. **3D**—Tetrahedral mesh −12 700 nodes, 67 000 elements. **Neonatal head**—108 000 nodes, 590 000 elements	DA homogenisation—assumes the AT has optical properties close to scalp	Imaging (DOT)	NR	NR
(Mahdy et al. 2022)	2022	COMSOL Multi-physics	FEM	Helmholtz equation	*q*- boundary absorption/impedance term. Between scalp and air: *q* = 0.16; between the skull and scalp: *q *= 0.39; between CSF and skull: *q* = 0.75; between GM and CSF: *q* = 0.44; between WM and GM: *q* = 0.5; and between tumour and WM: *q* = 0.5	150 mW	1000 and 1100	SDD of 4 mm	3D—5 rectangular layers	**Scalp**- 3. **Skull**- 7. **CSF**- 2. **GM**- 4. **WM**- 50. **Glioma and Meningioma**- 50 to 100	**1000 nm: Scalp**- 1.683; 0.033; 0.8; 1.37. **Skull**- 1.71; 0.022; 0.8; 1.45. **CSF**- 0.01; 0.45; 0.8; 1.33. **GM**- 0.521; 0.123; 0.8; 1.37. **WM**- 3.256; 0.176; 0.8; 1.37. **Glioma**- 0.3073; 0.044; 0.8; 1.37. **Meningioma**- 0.51392; 0.037; 0.8; 1.37 **1100 nm: Scalp**- 1.715; 0.019; 0.8; 1.37. **Skull**- 1.356; 0.016; 0.8; 1.45. **CSF**- 0; 0.1; 0.8; 1.33. **GM**- 0.528; 0.112; 0.8; 1.37. **WM**- 2.933; 0.157; 0.8; 1.37. **Glioma**- 0.2311; 0.045; 0.8; 1.37. **Meningioma**- 0.4052; 0.064; 0.8; 1.37	Triangular mesh	NR	Imaging (DOT)	NR	NR
(Dremin et al. 2022)	2022	COMSOL Multi-physics	FEM	Pennes’ Bio-heat Equation	NR	Gaussian beam. 50–250 mW (50 mW step). 10 min	760, 1064 and 1267	Perpendicular laser beam	3D—1 cylindrical layer	**Brain**- 5	**760 nm: Brain**- 6.47; 0.074; NR **1064 nm: Brain**- 4.54; 0.076; NR **1267 nm: Brain**- 3.79; 0.078; NR	Triangular mesh	NR	Thermal distribution	NR	NR
(Hirvi et al. 2023)	2023	Iso2mesh (mesh)	FEM	DE	Lipschitz BC—for the head as a domain. Dirichlet & Neumann BC—at the interfaces	Homogeneous diffuse emitter for photon injection	800	15 sources and 15 detectors. SDD between 7.5 and 55 mm	3D—prenatal head model (Collins-Jones atlas)	**Scalp and Skull**- 2. **CSF**- NR. **GM**- NR. **WM**- NR	**Scalp and Skull**- 16*; 0.015; 0.9; 1.4. **CSF**—1.6*; 0.004; 0.9; 1.4. **GM**- 5*; 0.048; 0.9; 1.4. **WM**- 10*; 0.037; 0.9; 1.4	Tetrahedral mesh—0.5 mm^3^ cubic voxels. Refined mesh around source and detector	Differences between the subarachnoid layer and sulci/ventricles were not considered	Imaging (DOT)	Compared with MC	Results are in good qualitative agreement between methods
(Borjkhani and Setarehdan 2023)	2023	MATLAB; COMSOL Multi-physics	FEM	DE	NR	3 mm diameter	NR	SDD of 40 mm	3D—1 rectangular layer. Inclusion under source and detector	**Brain**- 40. **Inclusion**- 2 mm side cube	**Brain**- 1.2*; 0.017; NR; 1.4. **Inclusion**- 5–6% change in optical properties	Triangular mesh	NR	Imaging (fNIRS)	Compared with analytical solution	Analytical solution is four orders of magnitude faster than FEM. FEM is better for complex head models

*AT* arachnoid trabeculae, *BC* boundary condition, *CSF* cerebrospinal fluid, *CT* computed tomography, *DA* diffusion approximation, *DE* diffusion equation, *DOT* diffuse optical tomography, *FEM* finite elements method, *FVM* finite volume method, *FDTD* finite-difference time-domain, *fNIRS* functional near-infrared spectroscopy, *GM* grey matter, *MC* Monte Carlo, *MRI* magnetic resonance imaging, *NIRS* near-infrared spectroscopy, *NR* not reported, *RTE* radiative transport equation, *SDD* source-detector distance, *tPBM* transcranial photobiomodulation, *UDDO* upwind-difference discrete-ordinates, *WM* white matter

*Scattering coefficient—in some instances, the scattering coefficient was reported instead of the reduced scattering coefficient

### Modelling conditions

For an overview of the core simulation parameters, the numerical methods, light propagation models/equations, and the boundary conditions will be detailed.

Regarding the **numerical methods**, FEM was the most used method, accounting for 79% of all records (*n* = 19). Three other numerical methods were identified, namely, the finite volume method (FVM), which was used in two articles, the finite-difference time-domain (FDTD), and the upwind-difference discrete-ordinates (UDDO), each used in one article.

For the **light propagation model**, there was a trend towards the use of the DE, being used in 63% of all records (*n* = 19), while the Helmholtz form of the DE was mentioned in 21% of records. It should be noted that, in one instance, although the Helmholtz equation was not mentioned, the equation described by the authors fitted the criteria. Nonetheless, it was considered an occurrence of the DE, per the authors’ description (Kannan and Przekwas 2012). Additionally, from the articles that used the DE, one combined it with the Pennes’ Bio-heat equation, and another with the Radiosity Transport Equation (RTE), a combination referred to as the Hybrid Radiosity-Diffusion Equation. Regarding the Pennes’ Bio-heat equation, it was used in three instances (including the one alongside the DE previously mentioned), for thermal distribution articles. Finally, the RTE was used solely in one article.

Concerning the **boundary conditions**, since some articles reported a combination of conditions, each combination was considered as one entry. Overall, the boundary conditions used are widely recognised, except for two articles which defined the conditions in the article (Guan et al. 2013; Mahdy et al. 2022) and two articles which did not describe the boundary conditions used in their studies (Dremin et al. 2022; Borjkhani and Setarehdan 2023). For more details on the conditions described in the article, refer to Table 1. More than half of the articles (63%, *n* = 19) used Robin boundary conditions (Bazrafkan and Kazemi 2014), either as a standalone or in combination with other conditions. Neumann and Dirichlet boundary conditions (Quarteroni and Valli 2008) were usually associated with Robin conditions and were once used together. Additionally, both Robin and Dirichlet conditions were used once in combination with other conditions described in the article (Zhou et al. 2019; Vera et al. 2020). Regarding boundaries that were only employed once, the Fresnel equations (Kannan and Przekwas 2012) and Lipschitz boundary conditions (Hirvi et al. 2023) have been utilised, the latter in combination with Dirichlet and Neumann conditions. It should be noted that in some instances, the authors did not name the conditions used; however, from the equations provided, it was possible to identify them, usually as Robin boundary conditions (Wang et al. 2010; Tanifuji et al. 2012; Sultan et al. 2013a, b, 2018; Bhattacharya and Dutta 2019; Vera et al. 2020).

**Fig. 2:**
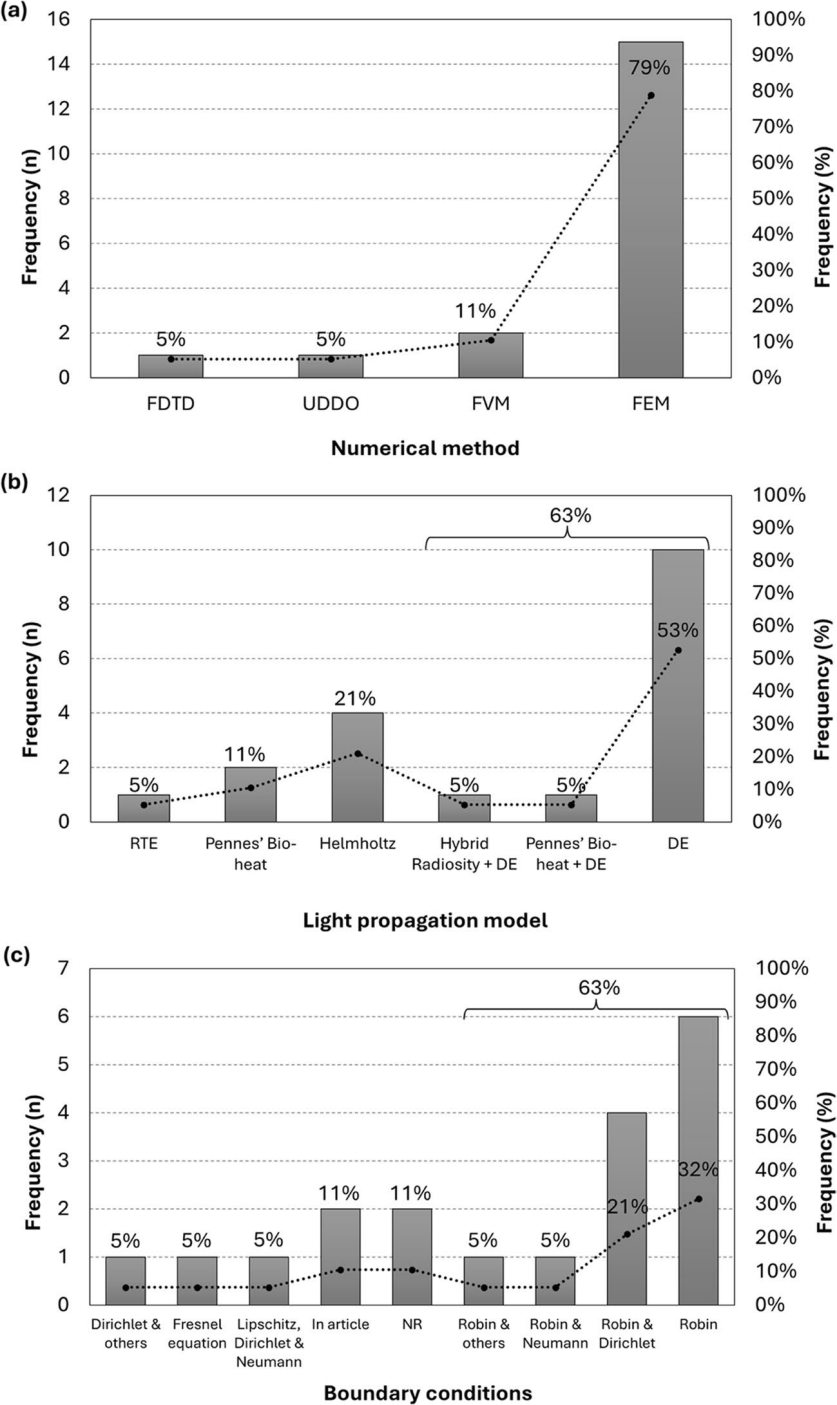
Numerical and relative frequency distribution of the numerical methods (**a**), the light propagation models (**b**), and the boundary conditions (**c**). *Abbreviations*: DE—diffusion equation; FEM—finite elements method; FVM—finite volume method; FDTD—finite-difference time-domain; NR—not reported; UDDO—upwind-difference discrete-ordinates

Figure 2 relates to a representation of the frequency distribution (numerical and relative frequencies) of the occurrences of the different numerical methods (Fig. 2a), light propagation models (Fig. 2b), and boundary conditions (Fig. 2c) used. In Fig. 2b, c, the sum of the relative frequencies referring to the use of the DE and Robin boundary conditions is represented, respectively.

### Tissue geometry and type

Concerning the tissues studied in the selected articles, their geometry, shape, and type of tissue were considered. For the **geometry**, namely, two-dimensional (2D) or 3D, there was a tendency towards 3D studies, relating to 68% of all records (*n* = 19, although three records included both 2D and 3D simulations). Regarding the **shape**, a distinction was made between articles which used a head model as a reference, e.g. Magnetic Resonance Imaging (MRI) scans, and those which considered each tissue as a geometric layer, i.e. horizontal layers, either rectangular or cylindrical. One article considered the tissues as concentric layers, with the outermost layer relating to the scalp, and the innermost to the brain (Lohrengel et al. 2021). Overall, the horizontal layers were more common, relating to 63% of all records, the rectangular layers (42%) being more frequent than the cylindrical (21%). For the articles that used head models as references, MRI scans were the most common (21%), followed by the Colin27 atlas (11%). The Collins–Jones model, the Shepp–Logan model, and computed tomography (CT) in combination with MRI images had one appearance each. Moreover, three articles used prenatal or neonatal heads as references for their studies, since this was their target demographic (Nourhashemi et al. 2016; Lohrengel et al. 2021; Hirvi et al. 2023).

**Fig. 3:**
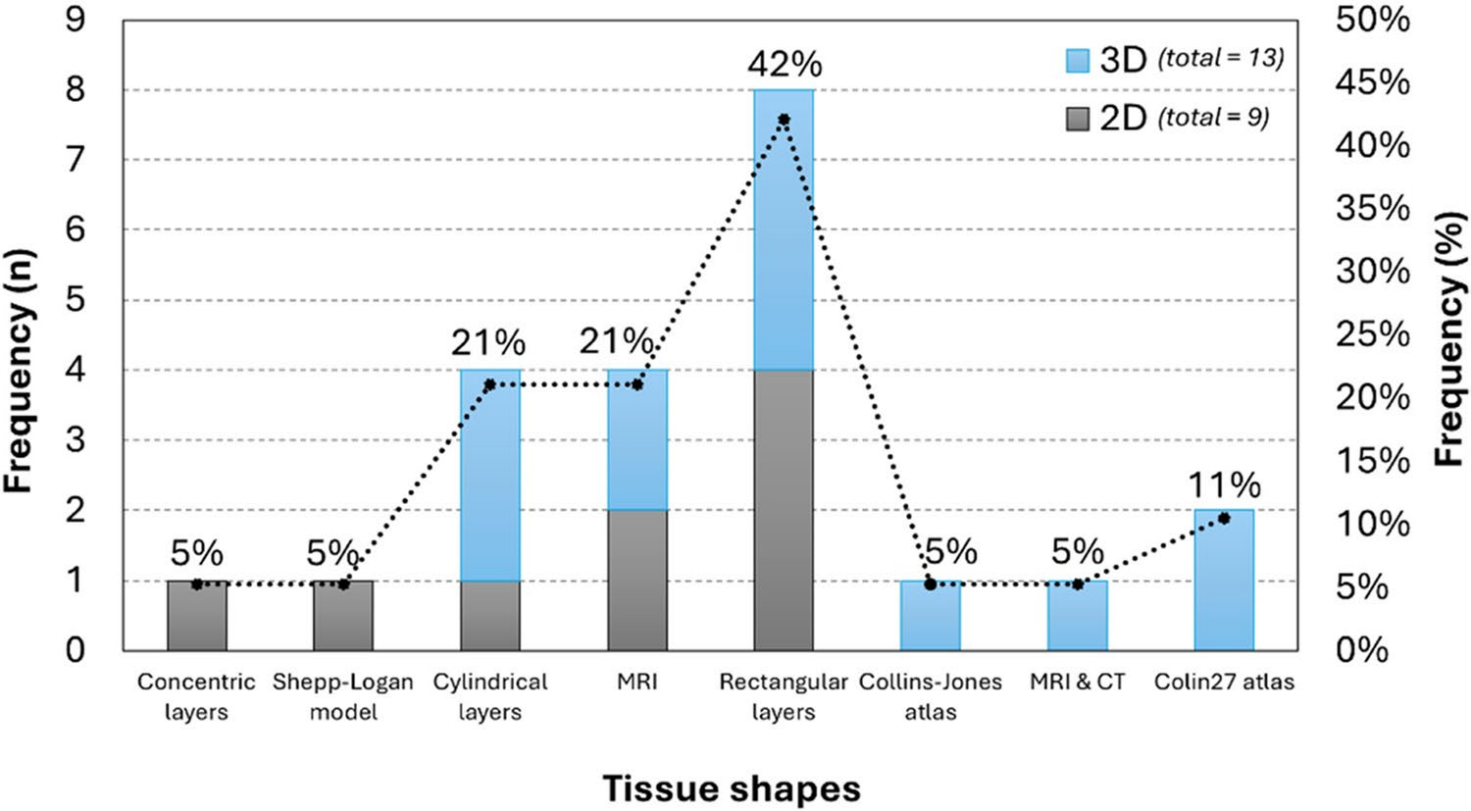
Numerical and relative frequency of the shapes considered for the models. Each shape is subdivided into 2D and 3D occurrences. *Abbreviations*: MRI—magnetic resonance imaging, CT—computed tomography

Figure 3 concerns the frequency distribution (numerical and relative frequencies) of the different shapes considered for the models, which are also subdivided by the number of 2D and 3D occurrences in each case.

For the **types of tissues** found in the included articles, since containing a simulation of the brain was an inclusion criterion, the brain (53%) or a combination of grey and white matter (47%) is found in every record (*n* = 19). For the outer layers, usually, there was a distinction between the scalp and the skull, but in 21% of records, the scalp and skull were considered as one layer. The scalp was usually followed by the skull, except for one article, where a layer of fat was considered between the two (Nourhashemi et al. 2016). This article was the only one which not only considered the fat tissue but also the dura mater, between the skull and the CSF. The latter was included in 63% of all records. Finally, some studies included specific types of tissue associated with, for example, neurological pathologies or spectroscopy studies, namely, inclusion (Lohrengel et al. 2021; Borjkhani and Setarehdan 2023), occlusion (Sultan et al. 2018), meningioma and glioma (Mahdy et al. 2022), and active region (Wang et al. 2010). Finally, one article studied the skin divided into three layers (epidermis, dermis, and subcutaneous tissue), but separately from the brain, and for this reason, these values were not included (Dremin et al. 2022).

**Fig. 4:**
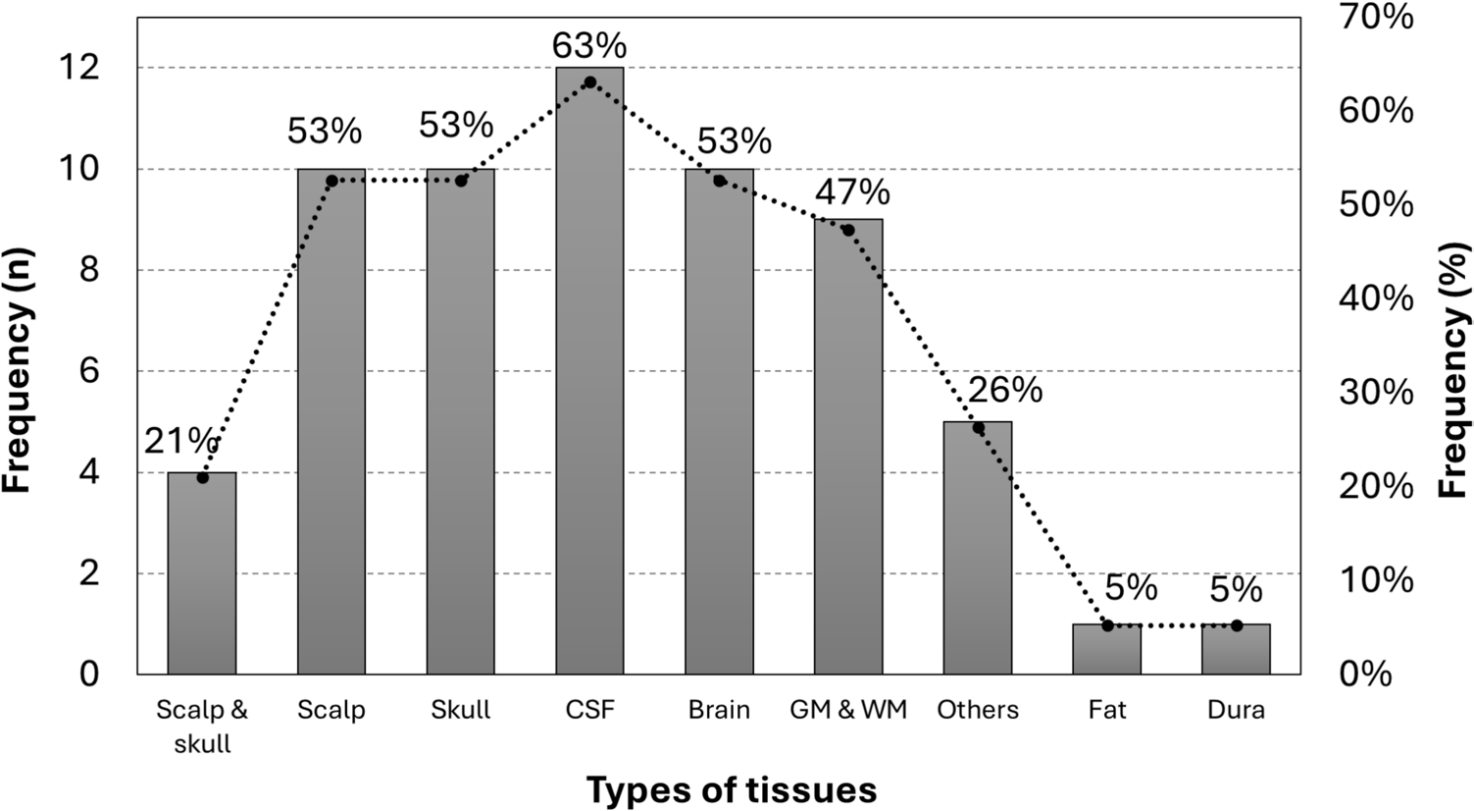
Numerical and relative frequency of the types of tissues. The category ‘Others’ refers to the inclusion, occlusion, meningioma, glioma, and active region tissues. *Abbreviations*: CSF—cerebrospinal fluid, GM and WM—grey matter and white matter

Figure 4 relates to the frequency distribution (numerical and relative frequencies) of the types of tissues found throughout the records.

There was some variation in tissues’ thickness throughout the several articles. Eight articles did not specify tissue thickness, usually articles which used head models as reference, except for two, which were geometric layers (Sultan et al. 2013a, b). Two articles included variation in tissue thickness, specifically for the scalp, skull, and CSF (Wang et al. 2010; Nourhashemi et al. 2016), and one for the cancerous tissue (Mahdy et al. 2022).

Table 2 contains a summary of the tissues’ thicknesses reported in the articles. For articles which included a variation of thickness, the median value was considered to calculate both average and standard deviation (STD).

**Table 2:** Reported thickness values for the head’s tissues

Tissue	Average (mm)	STD (mm)
Scalp and skull	5.50	3.50
Scalp	5.46	3.93
Fat	1.40	0.00
Skull	6.17	3.01
Dura	0.50	0.00
CSF	1.79	0.77
Brain	36.33	24.94
Grey matter	5.25	3.36
White matter	32.13	19.71
Inclusion	6.00	4.00
Occlusion	2.00	0.00
Glioma and meningioma	75.00	0.00

### Light parameters

Regarding the light parameters considered in the simulations, the light source was described as an isotropic light source, as a Gaussian beam, or not described at all, an equal number of times (21% each). The diameter and optical power of the source were sometimes described, in 16% and 11% of records, respectively. Concerning wavelength, five articles did not provide information, while the remaining ones examined wavelengths between 600 and 1267 nm. The most common wavelengths were in the 800 nm region (38%), followed by the 600 nm region (24%), with some overlap of wavelengths in articles which considered various wavelengths (*n* = 21).

Finally, for the light source and detector placement, the most commonly reported data was the separation between the source and detector (from 7.5 to 60 mm), and the depth at which the source is placed (usually reported as 1/*µ*_*s*_*’*).

### Tissue optical properties

Another significant aspect regarding the optical characterisation of the simulations is the optical properties of tissues. With the exception of one article, which did not provide this information (Sultan et al. 2013b), the reduced scattering coefficient or the scattering coefficient, and the absorption coefficient were always detailed, and in some instances, the anisotropy factor and the refractive index were also included.

**Fig. 5:**
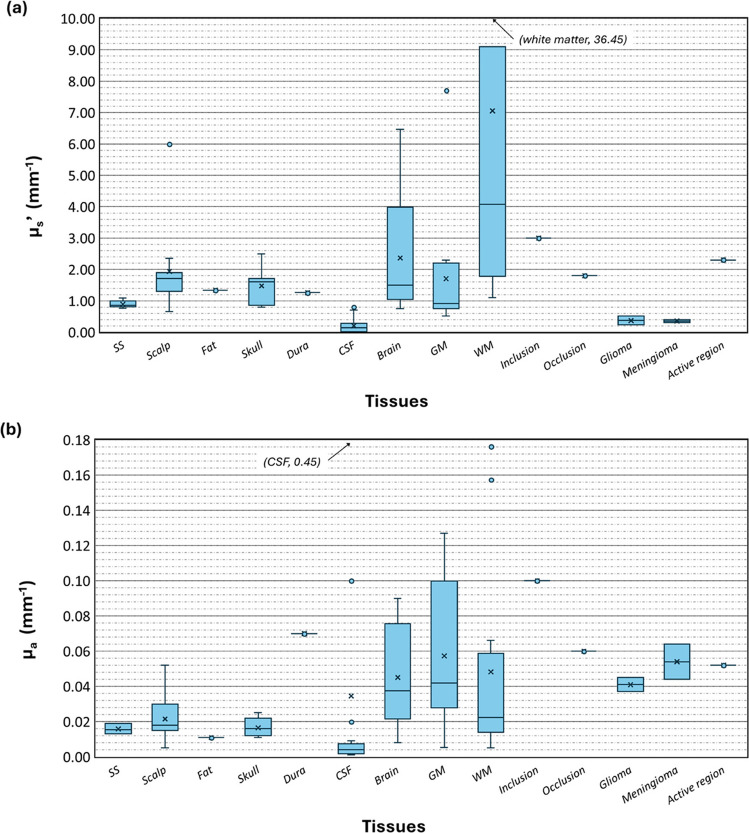
Representation of the distribution of tissues’ reduced scattering coefficients (*µ*_s_’) (**a**) and absorption coefficients (*µ*_a_) (**b**). One outlier in each plot (identified in the respective plot) was considered out of bounds, to improve comprehension of the plots. The fat, dura, inclusion, occlusion, and active region relate to only one value. *Abbreviations*: SS—scalp and skull, CSF—cerebrospinal fluid, GM—grey matter, WM—white matter

Figure 5 depicts a box-and-whisker plot for the *µ*_*s*_*'* (Fig. 5a) and *µ*_*a*_ (Fig. 5b) for all the head tissues. Although some articles mentioned the relation between wavelength and the optical properties above (Bhattacharya and Dutta 2019; Dremin et al. 2022; Mahdy et al. 2022), there is no clear tendency in how these parameters are affected by wavelength; therefore, all values were grouped. This will be further discussed in the ‘Challenges with simulating the head’s tissues’ section. Additionally, for articles which studied different properties for the same tissues, multiple entries were considered for the respective tissue and property. For the specific types of tissues, although inclusions were mentioned twice, one of the reports relates to a percentage of the brain’s properties and did not provide a specific value, so it was not included (Borjkhani and Setarehdan 2023).

For the anisotropy factor and the refractive index, only nine articles provided this information, with one of them only stating that *n* was constant (Vera et al. 2020), and another which studied how the *n* variation affected the simulation results, using more than one value, including an equation for a non-constant value (Guan et al. 2013). Additionally, as previously mentioned, the properties of the inclusion tissue were considered as a percentage of the brain properties in (Borjkhani and Setarehdan 2023). These values presented little variation for all tissues. Table 3 contains the average reported *g* and *n* values for each tissue, with the exception of the three articles abovementioned.

**Table 3:** Average of reported anisotropy factor (g) and refractive index (n)

Tissue	Average *g*	STD *g*	Average *n*	STD *n*
Scalp and skull	0.895	0.005	1.400	0.000
Scalp	0.850	0.050	1.465	0.095
Skull	0.850	0.050	1.505	0.055
Brain	0.850	0.050	1.480	0.080
CSF	0.873	0.042	1.380	0.054
Grey matter	0.863	0.045	1.385	0.015
White matter	0.863	0.045	1.385	0.015
Inclusion	0.900	0.000	NR	NR
Glioma	0.8	0.000	0.8	0.000
Meningioma	1.37	0.000	1.37	0.000

## Discussion

Overall, the records included in this review provide enough data to determine trends and limitations for these studies. However, some inconsistencies in the reporting of these studies can be pointed out in the light parameters category, where the description of the light source was neglected in some instances, and with some articles not referencing the wavelength for which the optical properties were considered. For this reason, there was not sufficient data in the reported light parameters for a significant analysis. Additionally, the boundary conditions description, which is a relevant parameter for the simulation, was lacking in two records. Nonetheless, since this was an infrequent occurrence (11% of records, *n* = 19), it did not impact the assessment of this parameter. There was some omission of the mesh description and simplifications employed; however, since these two parameters are more arbitrary, their omission does not necessarily impact the present analysis. The most limiting reporting inconsistency is in the description of tissues’ properties, specifically their thickness and optical properties, since the former is in some instances unreported, and the latter shows significant discrepancies. Thus, in this section, follows an analysis of not only the application of these studies, but also of their challenges, particularly for the tissues’ properties, as well as the validation methods used in the articles to support the respective findings.

### Fields of application of the numerical simulations

#### Imaging studies

This review found that most numerical studies of light propagation in the brain are made with the purpose of improving imaging techniques (74% of studies, *n* = 19). These simulations serve to validate whether proposed solutions can improve on current imaging models by assessing how different light source parameters and/or anthropometric features, or even pathologies (e.g. tumours, inclusions), can influence how light propagates through the head tissues. These factors affect depth sensitivity and spatial resolution, and by simulating these variations, it is possible to anticipate performance and enhance imaging algorithms prior to their application in clinical settings where time and error are critical.

These studies were categorised according to the characteristics of the simulation and the objectives outlined by the authors: studies focused on understanding the impact of tissue morphology on light propagation, studies aimed at detecting brain injury, and studies that evaluated the efficacy of numerical models in imaging algorithms.

Regarding **tissue morphology**, one study showed that increased skull thickness decreases the sensitivity of detection, while the opposite can be said about the CSF, up to 3 mm (Wang et al. 2010). Another studied the difference between using planar and non-planar boundaries between grey and white matter to represent the convolutions, sulci, and gyri of this tissue, concluding that a planar approach provides correct data at a low computational cost, but only if initial parameters are correct (Vera et al. 2020). Concerning the reference data for tissue morphology, one study compared the use of an atlas model or the subjects’ reference data and found that, although there were no major differences, using a reference model was a more significant source of error, concluding that using the subjects’ segmented anatomical model is preferable (Hirvi et al. 2023).

For how tissue morphology can affect **brain injury detection**, an article considered the presence of the arachnoid trabeculae (using properties similar to the scalp) in the CSF to improve the accuracy of light propagation without increasing computational complexity. This model showed increased sensitivity to abnormalities in the brain (Lohrengel et al. 2021).

Additionally, Mahdy et al*.* aimed to develop an algorithm for tumour detection by assessing differences in the distribution of spatial fluence rate, using infrared laser radiation, between brain and tumour tissue, obtaining positive results (Mahdy et al. 2022). For hematomas, the authors developed a simulation with the purpose of early detection of occlusions that was also able to detect changes in size, even for the smallest occlusion size, which was considered as 0.5 mm in diameter (Sultan et al. 2018); and for cerebral haemorrhage, an article aimed to accurately predict, detect, and quantify injury in the brain, using both the forward and inverse problem, and found that using the diffusion approximation (DE) led to relatively quick and accurate results (Kannan and Przekwas 2012).

Finally, one study focused on understanding the impact of using uniform and gradient refractive index in optical tomography obtained significantly different results, proving that this property cannot be ignored and considering its impact can affect diagnosis (Guan et al. 2013).

Six articles studied the efficiency of their **numerical models for improving imaging techniques**. Three articles compared their models with the Monte Carlo method. One showed a low percentage of errors and reduced computational time with FEM, stating it could serve as a strong replacement for the DE with Monte Carlo (Sultan et al. 2013b). The Hybrid Radiosity-Diffusion method was used in one article, which was commonly seen in articles prior to 2010, and showed great improvement over standard diffusion-based forward-problem solvers (Placati et al. 2016). The final study introduced the FDTD numerical method, obtaining results that agree well with Monte Carlo and the Hybrid Radiosity-Diffusion (Tanifuji et al. 2012).

The remaining three articles study photon propagation in the head tissues: one, which does not consider the CSF, obtains results similar to commercial brain phantoms (Sultan et al. 2013a); another confirms light is able to pass through the scalp, skull, and CSF, reaching the brain (Bazrafkan and Kazemi 2014); and one which compares FEM to an analytical approach, finding that although slower, FEM can better handle complex geometries (Borjkhani and Setarehdan 2023). All state that FEM is a fast and reliable method for these types of studies.

#### Thermal distribution studies

Tissue overheating is a common concern regarding the application of light in clinical practice. Over time, light exposure may result in tissue heating, which not only can lead to discomfort, but also impair the cognitive function of patients (Zhang et al. 2024). Additionally, imaging techniques can cause heat damage if the safety guidelines are not followed (Mittendorff et al. 2022). For photothermal therapies, even though there is an optimal level of tissue heating for their efficacy, there is a threshold after which there can be permanent tissue damage (Dremin et al. 2022; Yarmolenko et al. 2011).

To this end, four articles included an analysis of thermal variations due to the application of a light source in the head. In Bhattacharya and Dutta (2019), the authors intended to understand if the neuromodulatory effects of tPBM could be due to thermal variation. They found that for three wavelengths (630, 700, and 810 nm) the temperature in the brain tissue did not increase over 0.033 °C, which leads to the conclusion that thermal effects are unlikely. Another article studied the thermal impact of optical imaging techniques and found that temperature increased from 0.0025 to 0.26 °C at a depth of 15.9 mm in the adult brain, and from 0.03 to 2.85 °C at a depth of 4.9 mm in the neonatal brain (Nourhashemi et al. 2016).

One article that studied neural stimulation included the use of gold nanorods to increase thermal effects in the brain, reaching a temperature increase of 6 °C. It also demonstrated that the laser parameters that affect temperature distribution are pulse duration, laser power, and the absorption coefficient (Zhou et al. 2019). Finally, one article studied thermal variation to determine if it is possible to generate singlet oxygen to regulate the physiological functions of cells, simulating only one layer representing the brain, and reaching a temperature increase of 9.2 °C, the highest of all studies (Dremin et al. 2022).

These final two articles do not present comparable results to the first two since, due to the nature of their application, they do not consider the scalp or skull, with one of them even adding gold nanorods to enhance thermal effects, which justifies why their temperature variations are so much higher.

#### Transcranial photobiomodulation

Although all of the reviewed articles studied light propagation in tissue, only two focused on its application in transcranial photobiomodulation. This emerging therapy relates to the use of red to infrared light applied transcranially, so it becomes of interest to understand how the different light parameters and variations in anatomy may affect the light that reaches the brain, the target tissue of this therapy. Simulations are a straightforward alternative for these studies to understand how the different combinations of these factors affect light propagation, benefiting tPBM research.

One of these articles was mentioned in the previous section, as in addition to understanding how wavelength affects light penetration, it also included a thermal analysis to assert that tPBM effects are not thermally related. This study observed that 810 nm light has higher penetration than 630 and 700 nm, and that 0.2% of light was able to reach the grey matter (Bhattacharya and Dutta 2019). This study also simulated the absorption of water and four main chromophores of the grey matter, concluding that, in addition to water and lipid, the reduced and oxidised cytochrome c oxidase’s (CCO) absorption of NIR light is what causes light attenuation in the grey matter, which is thought to be the cause of tPBM’s effects (Salehpour et al. 2018).

By applying an evenly distributed multi-emitter array on the scalp, the other study aimed to improve light delivery to the brain by increasing the cerebral photon density while keeping each emitter operating within a safe thermal range, to take advantage of light scattering effects. The application of numerous sources resulted in a greater photon flux and penetration depth, indicating that it can enhance the delivery of transcranial NIR radiation. When compared to Monte Carlo, this method showed similar results (Yue and Humayun 2015).

### Challenges with simulating the head’s tissues

#### Simplification of tissue intricacies

Anatomical features for the feasibility of their studies. For example, not considering blood flow (Yue and Humayun 2015; Nourhashemi et al. 2016), and not considering the CSF (Sultan et al. 2013a, 2018). However, it can be pointed out that most articles simplified tissue geometry by considering horizontal rectangular or cylindrical layers, as opposed to studies which used imaging data as reference, obtaining more accurate representations of the tissues. Nonetheless, it is not evident from the literature if these geometrical simplifications are necessarily an issue.

One article which did not fit the inclusion criteria focused on studying the effect of the brain sulci and the presence of CSF in these spaces and concluded that the sulci had little difference in the final results, even though the presence of CSF increased the intensity of detected light (Okada et al. 1997). Furthermore, some of the studies included in this research examined the effects of morphological variations, previously described in the ‘Fields of application of the numerical simulations’ section. The following provides a concise overview of these findings. One study determined that using planar boundaries between grey and white matter provides similar data when compared to non-planar boundaries (Vera et al. 2020), another study compared the use of the subject’s own anatomical model versus a reference model and determined that the first is preferable (Hirvi et al. 2023), and the final study considered the impact of anatomical variations in skull thickness and learned that a higher skull thickness relates to a decreased sensitivity of detection (Wang et al. 2010). Besides the aforementioned studies that focus on how tissue morphology affects the simulation results, it is important to highlight the limited reported data on tissue thickness. This lack of information restricts the ability to perform a robust analysis on how this parameter affects simulation results. Given the significant morphological variability between subjects, further research is required to determine whether these morphological differences, namely, tissue thickness, play a relevant role when applying these simulations in a clinical context, or if certain approximations provide reliable results.

Regarding how physiological factors like blood flow or oxygenation affect light propagation in biological tissues, only one article considered blood perfusion in their model (Dremin et al. 2022). Although a complex factor to analyse, blood is known to absorb and scatter light (mainly due to haemoglobin, and depending on its oxygenation) (Bosschaart et al. 2014). Especially for studies of tPBM, in which the dose of light that reaches the brain is of interest, it is relevant to account for the influence of blood on the light propagation in the head.

Finally, another simplification that was noted in the reviewed articles was the grouping of tissues to reduce the amount of layers in the simulations. This happened mostly by grouping the scalp and skull in a single layer with homogenous optical properties, or by considering the brain as a single layer and not dividing it into grey and white matter, with the latter simplification being present in more than half the records. In this respect, it is important to consider that between the scalp and skull, there is a drastic change in the type of tissue and light interaction between their boundary that could play a significant role in differences between the two alternatives. For the brain, the difference in material between grey and white matter is more subtle, which can explain why grouping the tissues might be a good approximation.

However, there were three instances where other types of tissues were mentioned, namely, the meninges as a whole (Kannan and Przekwas 2012); the connective tissue between the arachnoid mater and pia mater, the arachnoid trabeculae (AT) (Lohrengel et al. 2021); and the fat and dura mater (Nourhashemi et al. 2016). In this last instance, both adult and neonatal head models were studied, and the inclusion of these tissues could be justified by the significant differences between them in both cases. However, the only one of these three articles that studied the impact of the inclusion of these tissues was the study of the AT, although they used a homogenisation algorithm to account for the AT among the CSF (Lohrengel et al. 2021). Thus, since most articles disregard these tissues, it should be determined if considering each of the meninges and fat versus using an overall approximation with the most relevant tissues (scalp, skull, CSF, brain) impacts the accuracy of the simulations in a way that is worth the computational cost. The omission of these tissues could be further justified by the fact that the optical properties considered for the fat tissue are in the same range as those of the scalp and skull as single layers. For the dura mater, this is only true for the reduced scattering coefficient, as the absorption coefficient is much higher than any value recorded for the scalp or skull. More experimental research that specifically addresses this issue is required.

#### Variation in reported optical properties

**Fig. 6:**
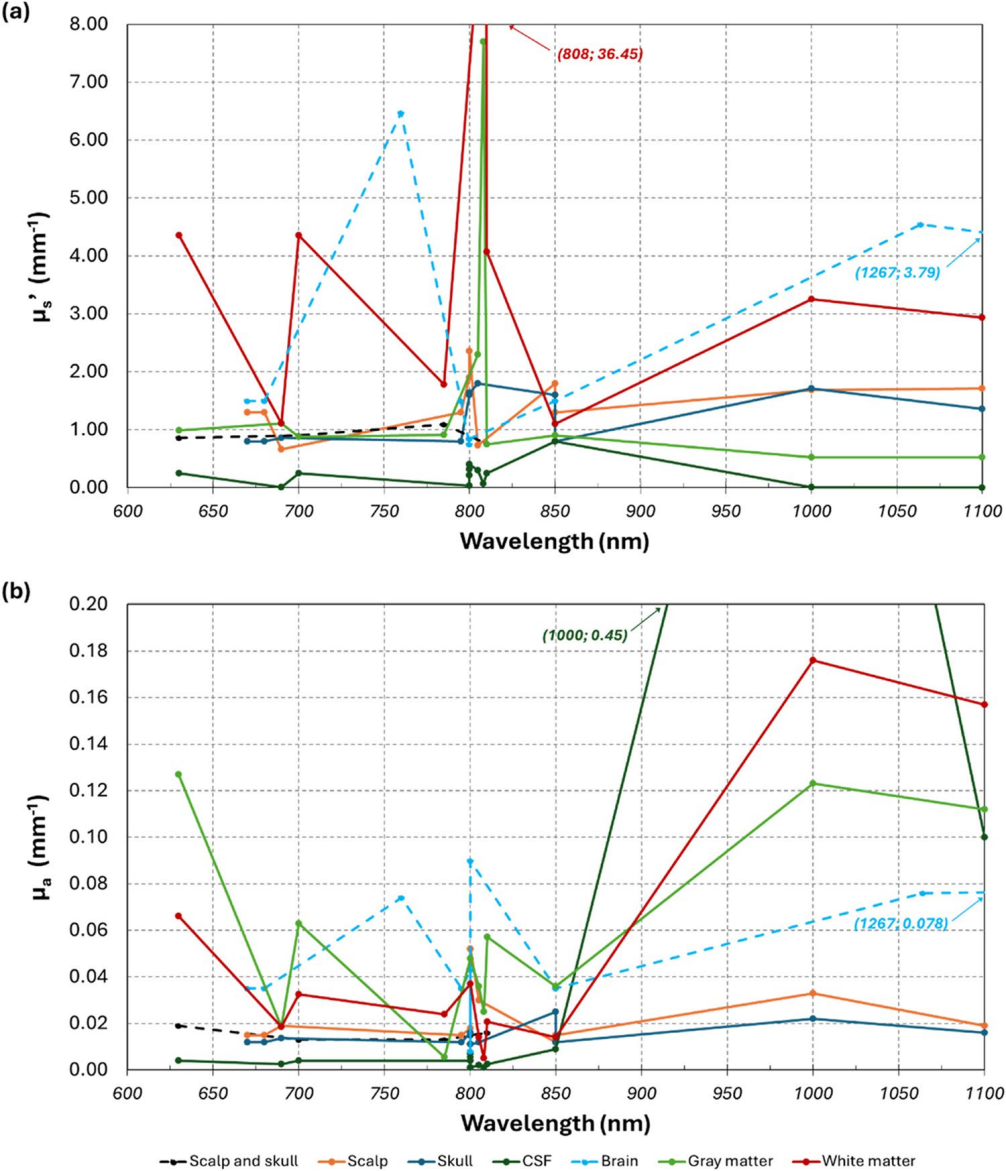
Variation of reduced scattering coefficients (*µ*_s_’) (**a**), and absorption coefficients (*µ*_a_) (**b**) with wavelength. Two points in each plot (identified in the respective plots) were considered out of bounds, to improve comprehension of the plots. *Abbreviations*: CSF—cerebrospinal fluid

One of the biggest variations found in these articles was the optical properties considered for each tissue. Although it is known that tissues exhibit different *µ*_*a*_ and *µ*_*s*_*’* for different wavelengths, in the values reported, there was no connection between the two. Values fluctuate throughout wavelengths without a tendency, with articles even providing different parameters for the same wavelength. Figure 6 illustrates this significant variation. Articles which did not provide wavelength information were not included. Two articles considered the same values for 680, 795, and 850 nm (Sultan et al. 2013a), and for 1000 and 1100 nm (Mahdy et al. 2022), and thus, each wavelength was considered as one separate entry. The inclusion, occlusion, active region, fat, and dura tissues were not included since they only exhibited values for one wavelength. Regarding the meningioma and glioma tissues, because their two values are from the same article and there is no data to compare them to, they were excluded (Mahdy et al. 2022).

Besides the optical properties relation to wavelength, considering the distribution of data represented on Fig. 5 (‘Tissue optical properties’ section), it is clear that the greatest discrepancies occur in both the *µ*_*a*_ and *µ*_*s*_*’* of the brain tissues (brain, grey matter, and white matter), and in the *µ*_*a*_ of the CSF. Additionally, regarding the reduced scattering coefficient, two articles stand out for using much higher values than the ones recorded in the remaining articles, namely, for the grey and white matter (Zhou et al. 2019), and for the scalp (Tanifuji et al. 2012). It could also be noted that the lowest recorded value for the scalp pertains to the article which included the meninges and scalp as one (Kannan and Przekwas 2012). Regarding the absorption coefficient, there were two evident outliers in the white matter, which were considered in the same article (Mahdy et al. 2022). The CSF presented three outliers, two used in the same article (Mahdy et al. 2022) and one in another (Tanifuji et al. 2012).

Due to the biological tissue variability, optical properties are not definite, and measured values can vary due to several factors, such as experimental procedure, subject variation, wavelength, and so forth (Jacques 2013; Ali and Bogdanovich 2023). Since the reviewed articles used different references for this data, fluctuations in values are expected. However, it is still uncertain to which extent using incorrect initial parameters can affect these simulations and if, in the future, personalised therapies will consider this fact to more accurately tailor treatment and diagnosis. Although these discrepancies in the CSF will be further discussed below, for the remaining tissues, specifically the brain tissues, these differences are not justified in any of the articles, and thus, apart from the aforementioned factors, there is no clear evidence for their occurrence.

Finally, for the refractive index and anisotropy factor, although less than half of the articles reported these values, there was very little discrepancy between values, as can be seen in Table 3 (‘Tissue optical properties’ section), showing that these are relatively well-established properties.

#### Simulating the CSF with the DA

For at least 20 years (Dehghani et al. 1999; Aydin et al. 2002), numerical simulation of the CSF was a subject of interest due to its low scattering and absorbing properties, not fulfilling the *µ*_*a*_/*µ*_*s*_*’* < < 1 ratio, causing doubts if it was valid to be simulated using the diffusion approximation (Lohrengel et al. 2021). Some articles that fitted most of the inclusion criteria, but were published before 2010, focused on solving the CSF as a clear non-scattering layer, using combinations of models such as the Hybrid Radiosity-Diffusion Equation (Ono et al. 2000) or the Hybrid Monte Carlo-Diffusion Method (Hayashi et al. 2003); otherwise, generally, the Diffusion Equation was used without considering the CSF (Leung et al. 2005). In the articles reviewed, this factor did not seem to hinder simulations. This may be because researchers are closer to identifying the optimal optical properties for which the CSF can be solved by the DA. Recently, *Lewis and Fang* provided updated insight in this topic and concluded that an accurate match to the gold standard Monte Carlo simulations is achieved when using a ${\mu {\prime}}_{s}$ of 0.15 mm^−1^ and maintain the ${\mu }_{a}$ at its physiological value (wavelength-dependent empirical value) (Lewis and Fang 2025).

### Validation of the deterministic numerical methods

Although it was possible to find several articles including a determinist numerical method for studies of light propagation in human head tissues, it became clear that Monte Carlo is still the most widely used simulation method for this purpose.

This results in a necessity to ascertain whether deterministic numerical methods are sufficiently efficient and accurate to serve as an alternative to the gold standard Monte Carlo methods. Although not every record included a validation for the numerical method used, more than half of the articles (12 out of the 19) compared the results obtained from the deterministic method to other methods, namely, Monte Carlo (Tanifuji et al. 2012; Yue and Humayun 2015; Nourhashemi et al. 2016; Placati et al. 2016; Bhattacharya and Dutta 2019; Hirvi et al. 2023), the Hybrid Radiosity-Diffusion (Tanifuji et al. 2012), the analytical solution (Sultan et al. 2013a, b; Borjkhani and Setarehdan 2023), and even experimental data (Sultan et al. 2013a, b; Guan et al. 2013; Sultan et al. 2018; Vera et al. 2020). In most cases, the results obtained through the method of interest showed small errors and were in agreement with the validating method. Additionally, one record, which used the FVM to solve the Hybrid Radiosity-Diffusion Equation, reported that the results showed improved computational efficiency and accuracy when compared to Monte Carlo (Placati et al. 2016); while another noted that, even though a quantitative comparison was not feasible, the methods have qualitative similarities (Yue and Humayun 2015).

However, one article stated that the analytical solution is faster than FEM, and thus, the use of FEM should be limited to more complex models which require this method (Borjkhani and Setarehdan 2023); another stated that CSF could produce erroneous results when using the DE (Bhattacharya and Dutta 2019), although this was discussed in the previous section; and finally, one article did not compare the experimental and simulation data, but rather used them complementarily to arrive at a conclusion (Vera et al. 2020).

In addition to these publications, several others point out that while Monte Carlo simulations are more accurate, they are also more time-consuming, and since the outcomes of numerical simulations are reliable, the difference in results does not justify the additional processing time required (Wang et al. 2010; Sultan et al. 2018). One article even states that, since Monte Carlo-based methods rely on the number of detected photons, they may be prone to errors related to a low signal-to-noise ratio (Hirvi et al. 2023).

This information aids in determining that deterministic numerical methods are constantly evolving, and that current solutions are able to produce relatively fast and accurate results, being a reliable tool for studying NIR light propagation in the human head.

**Fig. 7:**
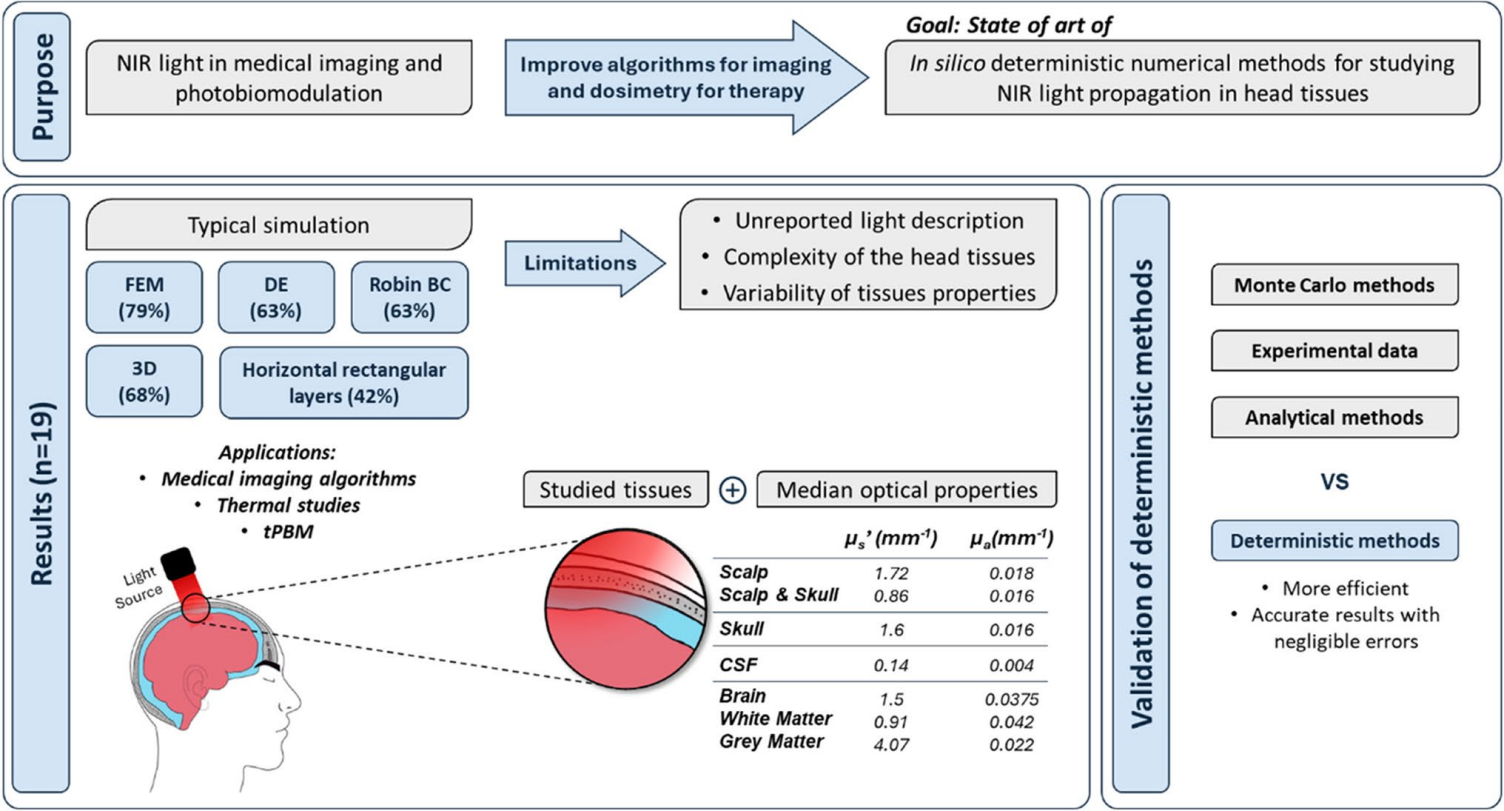
Summarisation of the aims and findings of the present research. *Abbreviations*: BC—boundary conditions, CSF—cerebrospinal fluid, DE—diffusion equation, tPBM—transcranial photobiomodulation, *µ*_s_’—reduced scattering coefficient, *µ*_a_—absorption coefficient

The topics herein discussed are schematized in Fig. 7.

## Conclusions

This literature review had the purpose to shed light on the state of the art of deterministic numerical simulations used for studying light propagation in head tissues, with particular interest in the scalp, skull, CSF, and brain. After following a systematic methodology for the collection and selection of relevant articles, it was possible to structure the collected data and attain useful knowledge and conclusions on this topic. A typical study of deterministic simulations of light propagation in head tissue can be designed despite the reported variability. A standard approach includes using FEM to solve the DE, applying Robin boundary conditions, and considering the geometry of tissues as 3D horizontal layers, each representing one of the following tissues: the scalp, skull, CSF, and brain or the grey and white matter.

Deterministic simulations are increasingly being considered as an alternative to the standard simulation methods, being employed in several medical optical fields, with the results of the screened articles showing that not only do they reach realistic results, but they are also more efficient, timewise, when compared with Monte Carlo. With several studies asserting that deterministic numerical models are a reliable alternative, it is foreseen that they will be more widely applied.

Since there is some inconsistency and variability in the reporting of light parameters and tissue properties, further studies are still required to understand if differences in tissue optical properties, and the simplification of the tissue morphology and biology, provide a reasonable estimate for these studies, or if more precision is required. Studies on how the use of the subject’s data can also contribute to improving knowledge on this topic.

## Supplementary Information

Below is the link to the electronic supplementary material.

Supplementary file1 (PDF 86.8 KB)

## Data Availability

No datasets were generated or analysed during the current study.
