# Supplementary material for: A contemporary systematic review on deterministic numerical simulations of light propagation in head tissues
Source: Biophys Rev. 2026 Jan 19;18(1):57–82. doi: 10.1007/s12551-025-01403-w (PMC13031484; doi:10.1007/s12551-025-01403-w)
Supplement: Supplementary file 1 — Supplementary file1 (PDF 86.8 KB) [file 12551_2025_1403_MOESM1_ESM.pdf]

Supplementary Table 1

Data base	Search Strategy
**PubMed**	((("numerical model"[Title/Abstract] OR "numerical modelling"[Title/Abstract] OR "numerical simulation"[Title/Abstract] OR "numerical methods"[Title/Abstract] OR "computer model"[Title/Abstract] OR "computer simulation"[Title/Abstract] OR "computer modelling"[Title/Abstract] OR "computational model"[Title/Abstract] OR "computational simulation"[Title/Abstract] OR "computational modelling"[Title/Abstract] OR "finite element"[Title/Abstract] OR "finite volume"[Title/Abstract] OR "discrete element"[Title/Abstract] OR "finite difference"[Title/Abstract] OR "ray tracing"[Title/Abstract] OR "beam propagation"[Title/Abstract]) AND (optical[Title/Abstract] OR light[Title/Abstract] OR photon[Title/Abstract] OR infrared[Title/Abstract] OR photo[Title/Abstract] OR photic[Title/Abstract] OR "low-level laser"[Title/Abstract] OR photobiomodulation[Title/Abstract])) AND (propagation[Title/Abstract] OR distribution[Title/Abstract] OR scattering[Title/Abstract] OR penetration[Title/Abstract] OR transmission[Title/Abstract] OR diffusion[Title/Abstract] OR transport[Title/Abstract] OR attenuation[Title/Abstract] OR reflectance[Title/Abstract] OR reflection[Title/Abstract] OR refraction[Title/Abstract] OR attenuation[Title/Abstract] OR absorption[Title/Abstract] OR diffraction[Title/Abstract])) AND (brain[Title/Abstract] OR cerebral[Title/Abstract] OR cortex[Title/Abstract] OR cerebrum[Title/Abstract] OR head[Title/Abstract] OR neuro[Title/Abstract])
**Scopus**	(TITLE-ABS-KEY ("numerical model" OR "numerical modelling" OR "numerical simulation" OR "numerical methods" OR "computer model" OR "computer simulation" OR "computer modelling" OR "computational model" OR "computational simulation" OR "computational modelling" OR "finite element" OR "finite volume" OR "discrete element" OR "finite difference" OR "ray tracing" OR "beam propagation") AND TITLE-ABS-KEY (optical OR light OR photon OR infrared OR photo OR photic OR "low-level laser" OR photobiomodulation) AND TITLE-ABS-KEY (propagation OR distribution OR scattering OR penetration OR transmission OR diffusion OR transport OR attenuation OR reflectance OR reflection OR refraction OR attenuation OR absorption OR diffraction) AND TITLE-ABS-KEY (brain OR cerebral OR cortex OR cerebrum OR head OR neuro))
**Web of science**	"numerical model" OR "numerical modelling" OR "numerical simulation" OR "numerical methods" OR "computer model" OR "computer simulation" OR "computer modelling" OR "computational model" OR "computational simulation" OR "computational modelling" OR "finite element" OR "finite volume" OR "discrete element" OR "finite difference" OR "ray tracing" OR "beam propagation" (Topic) and optical OR light OR photon OR infrared OR photo OR photic OR "low-level laser" OR photobiomodulation (Topic) and propagation OR distribution OR scattering OR penetration OR transmission OR diffusion OR transport OR attenuation OR reflectance OR reflection OR refraction OR attenuation OR absorption OR diffraction (Topic) and brain OR cerebral OR cortex OR cerebrum OR head OR neuro (Topic)
